# IL-1 blockade as a novel approach to treatment of hyperzincemia and hypercalprotectinemia, a possible new autoinflammatory syndrome

**DOI:** 10.1186/1546-0096-10-S1-A87

**Published:** 2012-07-13

**Authors:** Geraldina Lionetti, Jonathan A Bernstein, Dirk Holzinger, Michael Jeng, Johannes Roth, Neda Zadeh, Joyce Hsu

**Affiliations:** 1 Stanford University, Palo Alto, CA, USA; 2 University of Muenster, Muenster, Germany; 3

## Purpose

To describe a possible new autoinflammatory syndrome in a patient with hyperzincemia and hypercalprotectinemia with significant improvement upon initiation of anakinra.

## Methods

We describe a patient initially evaluated at 10 months of age for gross developmental delay, failure to thrive, splenomegaly, and microcytic anemia. Physical exam was significant for weight <3rd percentile, splenomegaly (down 5cm), and head lag. Initial laboratories revealed microcytic anemia (hemoglobin 8.7 g/dL); elevated platelets 499 K/uL, C-reactive protein 23.2 mg/dL (<0.2) and erythrocyte sedimentation rate 120 mm/h (0-10). Radiographs showed Erlenmeyer flask deformities of the femurs. Workup for lysosomal storage disorders, oncologic processes, and infections were negative. Genetic testing of known mutations in *FAS*, *CIAS1*, *ELA2*, *LPIN2*, *MVK*, *PSTPIP1*, and *TNFRSF1A* were negative. Immunoglobulins were elevated with normal T and B cells. On further workup for failure to thrive, a plasma zinc level was found elevated at 532 mcg/dL (60-120). Due to his persistently inflamed state, there was concern for hyperzincemia related to hypercalprotectinemia. Calprotectin level, measured by enzyme-linked immunosorbent assay, was elevated: 428,300 ng/mL (<420). On the basis of these findings the diagnosis of hyperzincemia associated with hypercalprotectinemia was made (OMIM 194470). Additionally, the patient’s workup has revealed the following mutations with unknown clinical significance: a heterozygous variant in the *MEFV* gene (T577A), a hemizygous duplication of exons 61-79 of the dystrophin gene (CPK normal), and a maternally inherited microduplication at Xp21.2. Colchicine was started due to the *MEFV* gene mutation with little to no improvement. Based on calprotectin’s role in inflammation, and possible link to IL-1, anakinra was initiated.

## Results

Four weeks after initiation of anakinra, our patient’s splenomegaly resolved, and inflammatory markers decreased significantly with improvement in his anemia.

## Conclusion

Approximately 8 patients with the diagnosis of hyperzincemia and hypercalprotectinemia have been described in the literature thus far, with the specific genetic cause still unknown. Consistent clinical findings are hepatosplenomegaly, anemia, elevated inflammatory markers, as well as characteristic elevations of serum zinc and calprotectin. Treatments have included corticosteroids and calcineurin inhibitors with little or no sustained benefit. We initiated anakinra due to the role of calprotectin in the inflammatory response. Calprotectin, a complex of S100A8 and S100A9 proteins, is in the family of calcium-binding proteins expressed by activated neutrophils and monocytes. It is unique in that it contains zinc-binding domains and leads to dysregulation of zinc levels when elevated. It is believed to be involved in antimicrobial activities and has a regulatory role in inflammatory reactions. Pro-inflammatory cytokines such as IL-1 have been shown to increase calprotectin levels. This is the first case report to show the use of an IL-1 inhibitor in a patient with hyperzincemia and hypercalprotectinemia.

## Disclosure

Geraldina Lionetti: None; Jonathan A. Bernstein: None; Dirk Holzinger: None; Michael Jeng: None; Johannes Roth: None; Neda Zadeh: None; Joyce Hsu: None.

